# Broadscale Ecological Patterns Are Robust to Use of Exact Sequence Variants versus Operational Taxonomic Units

**DOI:** 10.1128/mSphere.00148-18

**Published:** 2018-07-18

**Authors:** Sydney I. Glassman, Jennifer B. H. Martiny

**Affiliations:** a Department of Ecology and Evolutionary Biology, University of California—Irvine, Irvine, California, USA; b Department of Microbiology and Plant Pathology, University of California—Riverside, Riverside, California, USA; DOE Joint Genome Institute

**Keywords:** Illumina MiSeq, bacteria, exact sequence variants (ESVs), fungi, microbial ecology, operational taxonomic units (OTUs)

## Abstract

Recent discussion focuses on the best method for delineating microbial taxa, based on either exact sequence variants (ESVs) or traditional operational taxonomic units (OTUs) of marker gene sequences. We sought to test if the binning approach (ESVs versus 97% OTUs) affected the ecological conclusions of a large field study. The data set included sequences targeting all bacteria (16S rRNA) and fungi (internal transcribed spacer [ITS]), across multiple environments diverging markedly in abiotic conditions, over three collection times. Despite quantitative differences in microbial richness, we found that all α and β diversity metrics were highly positively correlated (*r* > 0.90) between samples analyzed with both approaches. Moreover, the community composition of the dominant taxa did not vary between approaches. Consequently, statistical inferences were nearly indistinguishable. Furthermore, ESVs only moderately increased the genetic resolution of fungal and bacterial diversity (1.3 and 2.1 times OTU richness, respectively). We conclude that for broadscale (e.g., all bacteria or all fungi) α and β diversity analyses, ESV or OTU methods will often reveal similar ecological results. Thus, while there are good reasons to employ ESVs, we need not question the validity of results based on OTUs.

**IMPORTANCE** Microbial ecologists have made exceptional improvements in our understanding of microbiomes in the last decade due to breakthroughs in sequencing technologies. These advances have wide-ranging implications for fields ranging from agriculture to human health. Due to limitations in databases, the majority of microbial ecology studies use a binning approach to approximate taxonomy based on DNA sequence similarity. There remains extensive debate on the best way to bin and approximate this taxonomy. Here we examine two popular approaches using a large field-based data set examining both bacteria and fungi and conclude that there are not major differences in the ecological outcomes. Thus, it appears that standard microbial community analyses are not overly sensitive to the particulars of binning approaches.

## Abstract (precis)

Microbial ecologists have made exceptional improvements in our understanding of microbiomes in the last decade due to breakthroughs in sequencing technologies. These advances have wide-ranging implications for fields ranging from agriculture to human health. Due to limitations in databases, the majority of microbial ecology studies use a binning approach to approximate taxonomy based on DNA sequence similarity. There remains extensive debate on the best way to bin and approximate this taxonomy. Here we examine two popular approaches using a large field-based data set examining both bacteria and fungi and conclude that there are not major differences in the ecological outcomes. Thus, it appears that standard microbial community analyses are not overly sensitive to the particulars of binning approaches.

## OBSERVATION

Characterization of microbial communities by amplicon sequencing introduces biases and errors at every step. Hence, choices concerning all aspects of molecular processing from DNA extraction method (1) to sequencing platform (2) are debated. Further downstream, the choices for computational processing of amplicon sequences are similarly deliberated (e.g., see references 3 to 5). Yet despite these ongoing debates, microbial ecology has made great strides toward characterizing and testing hypotheses in environmental and host-associated microbiomes (e.g., see references 6 and 7).

Within microbiome studies, operational taxonomic units (OTUs) have been used to delineate microbial taxa, as the majority of microbial diversity remains unrepresented in global databases (8). While any degree of sequence similarity could be used to denote individual taxa, a 97% sequence similarity cutoff became standard within microbial community analyses. This cutoff attempted to balance previous standards for defining microbial species (9) and recognition of spurious diversity accumulated through PCR and sequencing errors (10, 11).

Recently, it has been suggested that taxa should be defined based on exact nucleotide sequences of marker genes. Delineation of taxa by exact sequence variants (ESVs), also termed amplicon sequence variants (ASVs [12]) or zero-radius OTUs (zOTUs [13]), is not only expected to increase taxonomic resolution, but could also simplify comparisons across studies by eliminating the need for rebinning taxa when data sets are merged. Due to these advantages, there has been a surge in bioinformatic pipelines that seek to utilize ESVs and minimize specious sequence diversity (13–15). Moreover, some proponents have stated that ESVs should replace OTUs altogether (12). However, as with the adoption of any new approach, there remains a need to quantify how this new method compares to a large body of previous research. Furthermore, OTU classifications remain biologically useful for comparing diversity across large data sets (7) or identifying clades that share traits (16).

Here, we tested if use of ESVs versus 97% OTUs affected the ecological conclusions, including treatment effects and α and β diversity patterns, from a large field study of leaf litter communities. This study included a “site” and “inoculum” treatment, in which all microbial communities were reciprocally transplanted into all five sites (see Text S1 in the supplemental material) along an elevation gradient (17). We sequenced both bacteria (16S rRNA) and fungi (internal transcribed spacer 2 [ITS2]) from litterbags collected at three time points (6, 12, and 18 months after deployment) in separate sequencing runs. While we expected that the binning approach would alter observed richness, we hypothesized that it might not alter trends in α and β diversity, but that these results might differ based on the amplicon sequenced.

10.1128/mSphere.00148-18.1 TEXT S1  Supplemental methods. Detailed information on methods is shown. Download TEXT S1, PDF file, 0.1 MB. Copyright © 2018 Glassman and Martiny. 2018 Glassman and Martiny This content is distributed under the terms of the Creative Commons Attribution 4.0 International license.

In total, we analyzed >15 million bacterial and >20 million fungal sequences using UPARSE v10 (see Table S1 in the supplemental material), which allowed for a direct comparison of ESV versus 97% OTU approaches by keeping all other aspects of quality filtering and merging consistent (4). We selected a direct comparison with 97% OTUs as it is the most standard threshold and the clustering algorithms appear to be most effective at this level (R. Edgar, personal communication). A recent study also found that clustering thresholds from 87% to 99% yield highly stable results (18).

**FIG 1:**
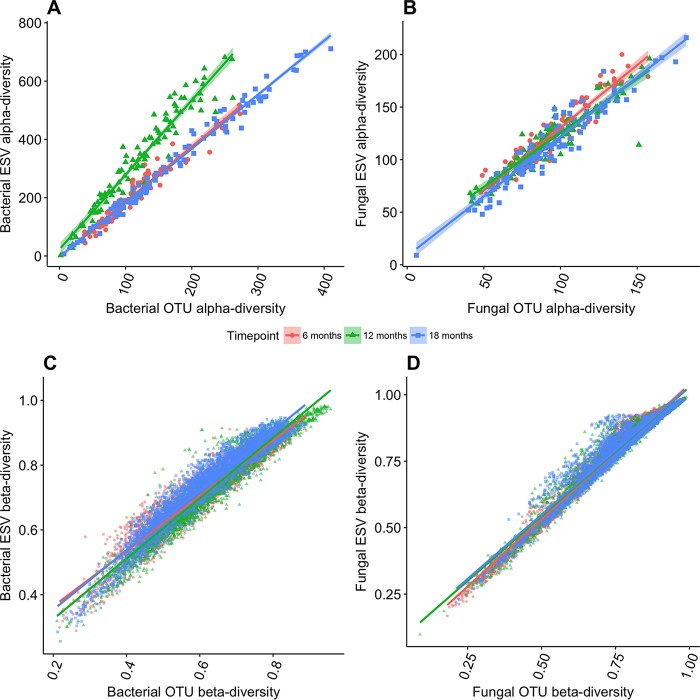
(A and B) Comparison of observed α diversity for (A) bacteria and (B) fungi as assayed by the richness of 97% similar operational taxonomic units (OTUs) versus exact sequence variants (ESVs). Numbers are total observed richness after normalizing to 10,000 sequences per sample from three time points (16, 12, and 18 months). (C and D) Comparison of observed β diversity for (C) bacteria and (D) fungi as assayed by the Bray-Curtis dissimilarity for OTUs versus ESVs from three time points (16, 12, 18 months).

ESV and OTU α diversity was strongly correlated across samples using four metrics for both bacteria and fungi (mean Pearson’s *r* = 0.95 ± 0.02; all *P* values are <0.001). For three metrics (Berger-Parker, Shannon, and Simpson), the ESV and OTU approaches were not only highly correlated (mean Pearson’s *r* = 0.95 ± 0.02), but nearly equivalent in their values (mean slope = 0.97) (see Table S2 in the supplemental material). For observed richness, ESV versus OTU was also highly correlated across all time points/sequencing runs (Pearson’s *r* > 0.92) (Fig. 1A and B). However, bacterial OTU richness was approximately half of ESV richness for the same sample (mean slope = 0.46), and fungal OTU richness was approximately three-quarters of ESV richness (mean slope = 0.79). We speculate that this difference between bacteria and fungi is due to the coarser phylogenetic breadth of the 16S versus ITS genetic regions.

10.1128/mSphere.00148-18.6 TABLE S1  Summary of data on Illumina MiSeq runs. Shown are the numbers of reads from each of the three different runs (corresponding to the three time points 6, 12, and 18 months), the number of samples, and the number of OTUs versus ESVs for fungal and bacterial amplicons. Download TABLE S1, DOCX file, 0.02 MB. Copyright © 2018 Glassman and Martiny. 2018 Glassman and Martiny This content is distributed under the terms of the Creative Commons Attribution 4.0 International license.

10.1128/mSphere.00148-18.7 TABLE S2  Pearson correlations and linear model relationships for 97% OTUs versus ESVs for α diversity and β diversity metrics for bacteria (16S rRNA) and fungi (ITS2) by time point. Download TABLE S2, DOCX file, 0.1 MB. Copyright © 2018 Glassman and Martiny. 2018 Glassman and Martiny This content is distributed under the terms of the Creative Commons Attribution 4.0 International license.

β diversity metrics were also strongly correlated across samples for ESVs and OTUs (Bray-Curtis average Mantel’s *r* = 0.96 for bacteria and 0.98 for fungi; all *P* values are <0.01 [Fig. 1C and D]), whether assessed by abundance-based (Bray-Curtis) or presence-absence (Jaccard) metrics (Table S2). Moreover, the values of the β diversity metrics were nearly identical regardless of binning approach (slopes of ~1).

**FIG 2:**
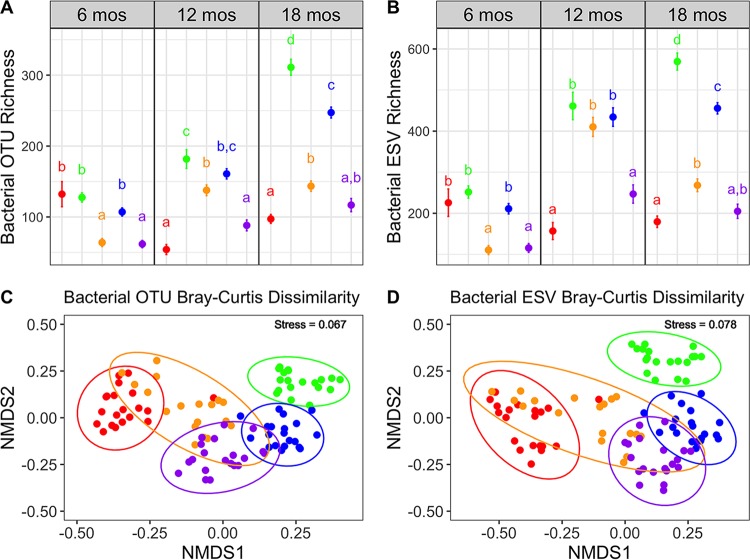
(A and B) Comparison of α diversity results using (A) operational taxonomic units (OTUs) versus (B) exact sequencing variants (ESVs) for bacteria across the elevation gradient at three time points (16, 12, and 18 months). Each point represents mean observed richness per litterbag per site, and lines indicated standard error (averaged across five inoculum treatments and four replicates; *n =* 20). Letters represent Tukey’s honestly significant difference (HSD) test significant differences across sites within a time point. (C and D) Comparison of β diversity results using nonmetric multidimensional scaling (NMDS) ordination of Bray-Curtis community dissimilarity of (C) bacterial OTUs and (D) bacterial ESVs colored by site at the final time point (18 months). Ellipses represent 95% confidence intervals around the centroid. Colors represent sites along the elevation gradient ranging from the lowest elevation (red = 275 m) to highest elevation (purple = 2,240 m), with middle elevation sites colored as follows: green = 470 m, orange = 1,280 m, and blue = 1,710 m.

The highly correlated α and β diversity metrics indicated that results based on these metrics should yield similar ecological conclusions. Indeed, the patterns of bacterial and fungal richness and community composition across the elevation gradient were nearly indistinguishable (Fig. 2; see Fig. S1 in the supplemental material), as were the statistical tests for both richness (see Table S3 in the supplemental material) and community composition (see Tables S4 and S5 in the supplemental material). Moreover, family- and genus-level compositions at each site along the gradient were virtually identical for bacteria (see Fig. S2 in the supplemental material) and highly similar for fungi (see Fig. S3 in the supplemental material), with no taxa being over- or underrepresented in the ESV versus OTU approaches for bacteria (Fig. S2C) and only one for fungi (Fig. S3C). We also included a mock community of eight distinct bacterial species in our PCR and sequencing runs. Both approaches resulted in highly similar mock community composition (see Fig. S4 in the supplemental material). Thus, we found no evidence that ESVs yield better taxonomic resolution or are more sensitive to detecting treatment effects (12). If anything, the ESV method appeared to be slightly less sensitive to detecting treatment effects on richness than the OTU method, especially for fungi in which fewer significant treatment effects were detected using ESVs (Table S3).

10.1128/mSphere.00148-18.2 FIG S1  (A and B) Comparison of α diversity results using (A) operational taxonomic units (OTUs) versus (B) exact sequencing variants (ESVs) for fungi across the elevation gradient at three time points (16, 12, and 18 months). Each point represents mean richness per litterbag per site (averaged across five inoculum treatments and four replicates; *n =* 20). Letters represent Tukey’s HSD test significant differences across sites within a time point. (C and D) Comparison of β diversity results using NMDS ordination of Bray-Curtis community dissimilarity of (C) fungal OTUs and (D) fungal ESVs colored by inoculum at the final time point (18 months). Ellipses represent 95% confidence intervals around the centroid. Colors represent inoculum from sites along the elevation gradient ranging from the lowest elevation (red = 275 m) to highest elevation (purple = 2,250 m). Download FIG S1, DOCX file, 0.1 MB. Copyright © 2018 Glassman and Martiny. 2018 Glassman and Martiny This content is distributed under the terms of the Creative Commons Attribution 4.0 International license.

10.1128/mSphere.00148-18.3 FIG S2  Relative abundance of bacterial sequences of the inoculum leaf litter from each of the five sites (mean of four samples per site) for OTUs versus ESVs summarized by (A) the 12 most abundant families or (B) the 12 most abundant genera. (C) Pearson correlations between mean relative abundance of all bacterial genera over 1% abundant in ESVs versus OTUs for inoculum leaf litter from the five sites. Points represent mean relative abundance, and lines represent standard errors for the four samples per site. Download FIG S2, DOCX file, 0.1 MB. Copyright © 2018 Glassman and Martiny. 2018 Glassman and Martiny This content is distributed under the terms of the Creative Commons Attribution 4.0 International license.

10.1128/mSphere.00148-18.4 FIG S3  Relative abundance of fungal sequences of the inoculum leaf litter from each of the five sites (mean of four samples per site) for OTUs versus ESVs summarized by (A) the 12 most abundant families or (B) the 12 most abundant genera. (C) Pearson correlations between mean relative abundance of all fungal genera over 1% abundant in ESVs versus OTUs for inoculum leaf litter from the five sites. Points represent mean relative abundance, and lines represent standard errors for the four samples per site. The most deviating point represents the fungal genus *Ascochyta* in the Ascomycete order Pleosporales. Download FIG S3, DOCX file, 0.1 MB. Copyright © 2018 Glassman and Martiny. 2018 Glassman and Martiny This content is distributed under the terms of the Creative Commons Attribution 4.0 International license.

10.1128/mSphere.00148-18.5 FIG S4  Relative abundance of bacterial sequences per mock community sample from each Illumina MiSeq run for each time point with either ESVs or 97% OTUs. The three time points represent 6 months (T1), 12 months (T2), and 18 months (T3) after transplantation. Both ESVs and OTUs largely recapitulated the eight mock community taxa at the family level, although in both cases more than 8 taxa were found. Bacterial taxa included in the mock community were Bacillus subtilis (F. *Bacillaceae*), Escherichia coli (F. *Enterobacteriaceae*), Salmonella enterica (F. *Enterobacteriaceae*), Enterococcus faecalis (F. *Enterococcaceae*), Lactobacillus fermentum (F. *Lactobacillaceae*), Pseudomonas aeruginosa (F. *Pseudomonadaceae*), Staphylococcus aureus (F. *Staphylococcaceae*), and Listeria monocytogenes (F. *Listeriaceae*). For ESVs, there were more taxa within each of these dominant families than for OTUs, but since all the taxa included in the mock community have multiple copies of 16S rRNA, it is unclear if these are truly spurious taxa or represent real genetic variation within the 16S rRNA gene. (For information on taxa in the mock community, see https://www.zymoresearch.com/zymobiomics-community-standard.) For both OTUs and ESVs, there were low-abundance sequences from families common in the experimental samples (i.e., *Oxalobacteraceae*), and these likely represent spillover between the bar-coded samples. Download FIG S4, DOCX file, 0.03 MB. Copyright © 2018 Glassman and Martiny. 2018 Glassman and Martiny This content is distributed under the terms of the Creative Commons Attribution 4.0 International license.

10.1128/mSphere.00148-18.8 TABLE S3  Analysis of variance (ANOVA) results for OTUs and ESVs at each of the three time points, testing for effects of site and inoculum and their interaction on bacterial or fungal richness. Download TABLE S3, DOCX file, 0.03 MB. Copyright © 2018 Glassman and Martiny. 2018 Glassman and Martiny This content is distributed under the terms of the Creative Commons Attribution 4.0 International license.

10.1128/mSphere.00148-18.9 TABLE S4  Permutational multivariate ANOVA (PERMANOVA) results for bacterial OTUs versus ESVs, testing for effects of site and inoculum and their interaction on Bray-Curtis community dissimilarity (data plotted in Fig. 2C and D). Download TABLE S4, DOCX file, 0.03 MB. Copyright © 2018 Glassman and Martiny. 2018 Glassman and Martiny This content is distributed under the terms of the Creative Commons Attribution 4.0 International license.

10.1128/mSphere.00148-18.10 TABLE S5  PERMANOVA results for fungal OTUs versus ESVs, testing for effects of site and inoculum and their interaction on Bray-Curtis community dissimilarity (data plotted in Fig. S1C and D). Download TABLE S5, DOCX file, 0.03 MB. Copyright © 2018 Glassman and Martiny. 2018 Glassman and Martiny This content is distributed under the terms of the Creative Commons Attribution 4.0 International license.

Despite quantitative differences in microbial richness, ecological interpretation of our large bacterial and fungal community data set was robust to the use of ESVs versus 97% OTUs. Thus, even though there are good reasons to take an ESV approach, we need not question the validity of ecological results based on OTUs. Indeed, while previous studies have found that ESVs can help explain additional variation among samples (19, 20), the α and β diversity patterns of ESVs and OTUs in these studies were also quite similar. In general, we suspect that the robustness of such comparisons will vary depending on the breadth of the microbial community targeted. For instance, here we characterized all bacteria and fungi in a diverse environmental community, as opposed to a narrower subset of taxa or a less diverse, host-associated community.

Finally, both 97% OTUs and ESVs mask ecologically important trait variation of individual taxa (19, 21). In our study, ESVs only slightly increased the detection of fungal and bacterial diversity (1.3 and 2.1 times OTU richness, respectively), highlighting that ribosomal marker genes at any resolution are generally poor targets for improving genetic resolution within a microbial community. For example, it is widely known that many taxa can share the same 16S rRNA (21) or ITS (22). Thus, if strain identification is critical, then a full genome (21) or amplicon of a less conservative marker gene (23) is required. However, for broadscale community α and β diversity patterns, although the vagaries of molecular and bioinformatics processing inevitably add noise to microbial sequencing data, strong community-level signals will likely emerge with suitable study designs and statistics regardless of binning approach.

### Data availability.

Sequences were submitted to the National Center for Biotechnology Information Sequence Read Archive under accession no. SRP150375 and BioProject no. PRJNA474008. All data and scripts to recreate all figures and statistics from this article can be found on github at https://github.com/sydneyg/OTUvESV.
