# Supplementary material for: Broadscale Ecological Patterns Are Robust to Use of Exact Sequence Variants versus Operational Taxonomic Units
Source: mSphere. 2018 Jul 18;3(4):e00148-18. doi: 10.1128/mSphere.00148-18 (PMC6052340; doi:10.1128/mSphere.00148-18)
Supplement: TEXT S1 [file sph004182596s1.pdf]

Supplemental Text S1

Title: Broadscale ecological patterns are robust to use of exact sequence variants versus operational taxonomic units

Authors: Sydney I. Glassman and Jennifer B. H. Martiny

Affiliations: Department of Ecology and Evolutionary Biology, UC Irvine, and Department of Microbiology and Plant Pathology, UC Riverside

Corresponding author e-mail: sydney.glassman@ucr.edu

Methods:

The dataset is derived from a reciprocal transplant experiment conducted with leaf litter across five sites ranging in elevation from 275 to 2240m in southern California, USA (Glassman et al in prep). Precipitation and temperature co-vary along this elevation gradient (275m, 470m, 1280m, 1710m, 2240m). Total precipitation throughout the duration of the experiment ranged from 213 to 1415mm and mean soil temperature ranged from 11 to 26 °C. As part of the transplant, we constructed microbial litterbags with 0.22 μm nylon mesh that allow for movement of water and nutrients but prevent dispersal of microbes (1). We filled each bag with a common substrate of 5g of homogenized, gamma-irradiated, ground-up litter from a middle elevation site. We then inoculated each bag with 50mg of homogenized, ground-up litter containing the natural microbial community from each site for the initial "inoculum". At each site along the gradient, we deployed the bags into 4 replicate plots in October 2015, and collected samples at 6, 12, and 18 months until April 2017. DNA was extracted from 100 litterbags (5 sites x 5 inoculum treatments x 4 plots) at each of the three time points (n=300) using the FastDNA Spin Kit for Soil (MP Biomedicals, Santa Ana, CA, USA) following the manual with the modification of adding three freeze/thaw cycles (30s in liquid Nitrogen followed by 3-5 min in 60°C water bath) prior to bead beating step to improve cell lysis.

To characterize bacterial composition, we amplified the V4 region of the 16S ribosomal RNA (rRNA) gene using the 515F-926R primers (2) with modifications to improve diversity (3) with the forward primer as the bar-coded primer. PCR mixtures for amplification contained 0.2 μL of NEB Hot Start Taq (5 units/μL) DNA polymerase (New England Biolabs, Ipswich, MA, USA), 2.5 μL of 10× 5Prime Hotmaster buffer minus $MgCl_2$ (Quantabio, Beverly, MA, USA), 0.6 μL $MgCl_2$ (50 mM), 0.50 μL dNTPs (10mM), 0.50 μL of 10 μM non-barcoded primer, 5 μL of 1 μM barcoded primer, 0.25 μL of BSA (20mg/ml), 5 μL of DNA template (diluted to 1:10 or 1:50 to overcome inhibitors), and water up to 25 μL. PCR conditions were as follows: denaturation at 94°C for 3 min; 35 amplification cycles of 45 s at 94°C, 30 s at 55°C, 20 s at 68°C, followed by a 10-min final extension at 68°C. For the 16S libraries, we included a mock community of eight bacteria strains from Zymo Microbiomics (Zymo Research, Irvine, CA, USA) that we PCR amplified and included in each sequencing run.

To characterize fungal composition, the ITS2 region of the Internal Transcribed Spacer (ITS) was amplified using the ITS9f-ITS4 primer combination designed by (4) and modified for Illumina MiSeq (5) following a staggered design (6). PCR mixtures for

amplification contained 21.5 µl Platinum PCR Supermix (1.1x, Thermo Scientific, Waltham, MA, USA), 1 µl BSA (10 mg/ml, NEB, Ipswich, MA), 0.75 µl of both primers (10 µM, ITS9f and barcoded ITS4) and 1 µl of DNA template (diluted to 1:10) for a 25 µL reaction. PCR conditions were as follows: denaturation at 94°C for 5 min; 35 cycles of 45 s at 95°C, 1min at 50°C, 90 s at 72°C, followed by a 10-min final extension at 72°C.

PCR products were pooled visually according to intensity of bands based on electrophoresis gel images (scaled as weak, medium, or strong bands). Samples were pooled into six separate libraries (3 time points for each amplicon with 100-150 samples each). The pooled libraries were then purified according to the AMPure XP magnetic Bead protocol (Beckman Coulter Inc, Brea, CA, USA). For 16S, AMPure magnetic beads were used. For ITS2, we followed the same protocol but instead used a homemade solution of Sera-mag SpeedBeads (Fisher Scientific). The purified libraries were quality checked with an Agilent BioAnalyzer 2100 at the UCI Genomics High-Throughput Facility (UC Irvine, CA, USA) for size and concentration. The libraries were then sequenced in six separate Illumina MiSeq PE runs (2 x 250 bp) at the DNA Technologies Core, UC Davis Genome Center, Davis, CA, USA. Sequences were submitted to the National Center for Biotechnology Information Sequence Read Archive under accession number SRP150375.

All bioinformatics processing was conducted in UPARSE (7) version 10 (https://www.drive5.com/usearch/manual/uparse_pipeline.html). We processed each amplicon library by each timepoint in order to examine variation in patterns among sequencing runs. We chose the UPARSE pipeline for ease of comparison of OTU versus ESV methods while keeping all other aspects of quality filtering and merging the same (7). It is possible that results might differ if an alternative pipeline such as DADA2 (8) or Deblur (9) was chosen for generating ESVs. Evidence suggests that ESV richness might have been lower if we had used DADA2 (http://fiererlab.org/2017/05/02/lumping-versus-splitting-is-it-time-for-microbial-ecologists-to-abandon-otus/). However, we think it unlikely that the general pattern of high correlations of alpha- and beta-diversity amongst varying approaches would differ given the robustness of our results and other recent findings that a wide range of sequence similarity cutoffs does not affect broad-scale ecological outcomes (10).

First, primers were stripped, then reads were truncated based on quality, forward and reverse reads were merged, and then merged pairs were quality filtered using the fastq_filter command with a fastq_maxee parameter of 1.0. Next, at the same point in the pipeline, UPARSE can process both 97% OTUs with the "-cluster_otus" command and ESVs with the "unoise3" command. We used the default settings for each function, which for OTUs removes singletons with the "minsize 2" parameter, and the default minimum of 8 sequences per cluster for "unoise3". Both of these methods are open reference and thus capture novel diversity. OTU tables were made with the otutab command for both 97% OTUs and ESVs.

Taxonomy was assigned in QIIME 1.9 (11) using the assign_taxonomy.py command. For 16S, assignments were made with the Greengenes database (12) using rdp classifier and 0.80 similarity cutoff. For ITS2, we used the UNITE database (13), accessed on June 28, 2017, using blast and minimum E value of 0.001. For ITS2, only

reads mapping to kingdom Fungi were retained, and for 16S, all reads mapping to chloroplasts, mitochondria, or unclassified were removed.

Preliminary alpha diversity analyses were conducted in UPARSE. Samples were normalized to 10,000 sequences per sample using the otutab_norm command and alpha diversity metrics were calculated on the subsampled OTU tables using the alpha_div command. Next, these richness metrics were imported to R (14), where we performed Pearson correlations and linear regressions to determine the correlation, intercept, and slope of the relationship between four separate alpha diversity metrics (Berger-Parker, Observed Richness, Shannon, Simpson) for both the OTU and ESV approaches for each amplicon and at each time point. We then performed ANOVA and post-hoc Tukey HSD tests to determine if the significance of our treatments on observed richness were different for OTU versus ESV for each time point for each amplicon. All figures were made using the ggplot2 package (15) in the R software environment.

For beta diversity analyses, we took the raw OTU and ESV tables from UPARSE and calculated Bray-Curtis and Jaccard dissimilarity matrices for bacteria and fungi at each time point in the R package vegan (16) using the avgdist function (https://github.com/vegandevs/vegan/blob/master/man/avgdist.Rd). Specifically, we used this function to calculate a median, square-root transformed, Bray-Curtis or Jaccard dissimilarity matrix based on 100 subsamples of either 7,000 seq/sample for bacteria or 17,000 seq/sample for fungi. We then ran Mantel correlations in vegan to test the correlation between beta-diversity metrics between the binning methods for bacteria in fungi. We visualized these correlation in ggplot. Next, PERMANOVA tests were conducted with the Adonis function in vegan to test the effects of our two treatments and their interactions on the Bray-Curtis community dissimilarity of both bacteria and fungi as assessed by either OTUs or ESVs. We then visualized these patterns with NMDS using the metaMDS function in vegan.

To determine if binning method (OTUs vs ESVs) affected distribution of taxonomic groups among each site, we summarized the top 12 most abundant families or genera at each site for the inoculum leaf litter using the dplyr package and visualized this information with barplots the ggplot2 package in R. All data and scripts to re-create all figures and statistics from paper can be found on github: https://github.com/sydneyg/OTUvESV.

References

1. Allison SD, Lu Y, Weihe C, Goulden ML, Martiny AC, Treseder KK, Martiny JBH. 2013. Microbial abundance and composition influence litter decomposition response to environmental change. Ecology 94:714-725.
2. Caporaso JG, Lauber CL, Walters WA, Berg-Lyons D, Huntley J, Fierer N, Owens SM, Betley J, Fraser L, Bauer M, Gormley N, Gilbert JA, Smith G, Knight R. 2012. Ultra-high-throughput microbial community analysis on the Illumina HiSeq and MiSeq platforms. Isme Journal 6:1621-1624.
3. Apprill A, McNally S, Parsons R, Weber L. 2015. Minor revision to V4 region SSU rRNA 806R gene primer greatly increases detection of SAR11 bacterioplankton. Aquatic Microbial Ecology 75:129-137.

4. Ihrmark K, Bodeker ITM, Cruz-Martinez K, Friberg H, Kubartova A, Schenck J, Strid Y, Stenlid J, Brandstrom-Durling M, Clemmensen KE, Lindahl BD. 2012. New primers to amplify the fungal ITS2 region - evaluation by 454-sequencing of artificial and natural communities. Fems Microbiology Ecology 82:666-677.
5. Looby CI, Maltz MR, Treseder KK. 2016. Belowground responses to elevation in a changing cloud forest. Ecology and Evolution 6:1996-2009.
6. Tremblay J, Singh K, Fern A, Kirton ES, He SM, Woyke T, Lee J, Chen F, Dangl JL, Tringe SG. 2015. Primer and platform effects on 16S rRNA tag sequencing. Frontiers in Microbiology 6.
7. Edgar RC. 2013. UPARSE: highly accurate OTU sequences from microbial amplicon reads. Nature Methods 10:996-998.
8. Callahan BJ, McMurdie PJ, Rosen MJ, Han AW, Johnson AJA, Holmes SP. 2016. DADA2: High-resolution sample inference from Illumina amplicon data. Nature Methods 13:581-+.
9. Amir A, McDonald D, Navas-Molina JA, Kopylova E, Morton JT, Xu ZZ, Kightley EP, Thompson LR, Hyde ER, Gonzalez A, Knight R. 2017. Deblur Rapidly Resolves Single-Nucleotide Community Sequence Patterns. Msystems 2.
10. Botnen SS, Davey ML, Halvorsen R, Kauserud H. 2018. Sequence clustering threshold has little effect on the recovery of microbial community structure. Molecular Ecology Resources doi:https://doi.org/10.1111/1755-0998.12894.
11. Caporaso JG, Kuczynski J, Stombaugh J, Bittinger K, Bushman FD, Costello EK, Fierer N, Pena AG, Goodrich JK, Gordon JI, Huttley GA, Kelley ST, Knights D, Koenig JE, Ley RE, Lozupone CA, McDonald D, Muegge BD, Pirrung M, Reeder J, Sevinsky JR, Tumbaugh PJ, Walters WA, Widmann J, Yatsunenko T, Zaneveld J, Knight R. 2010. QIIME allows analysis of high-throughput community sequencing data. Nature Methods 7:335-336.
12. DeSantis TZ, Hugenholtz P, Larsen N, Rojas M, Brodie EL, Keller K, Huber T, Dalevi D, Hu P, Andersen GL. 2006. Greengenes, a chimera-checked 16S rRNA gene database and workbench compatible with ARB. Applied and Environmental Microbiology 72:5069-5072.
13. Koljalg U, Larsson KH, Abarenkov K, Nilsson RH, Alexander IJ, Eberhardt U, Erland S, Hoiland K, Kjoller R, Larsson E, Pennanen T, Sen R, Taylor AFS, Tedersoo L, Vralstad T, Ursing BM. 2005. UNITE: a database providing web-based methods for the molecular identification of ectomycorrhizal fungi. New Phytologist 166:1063-1068.
14. R Core Team. 2017. R: A Language and Environment for Statistical Computing, R Foundation for Statistical Computing, Vienna, Austria. http://www.r-project.org/.
15. Wickham H. 2009. ggplot2: Elegant Graphics for Data Analysis. Springer-Verlag New York.
16. Oksanen J, Blanchet F, Kindt R, Legendre P, Minchin P, O'Hara R, Simpson G, Solymos P, Stevens M, Wagner H. 2012. vegan: Community Ecology Package. R package version 2.0-10.
