# Supplementary material for: Broadscale Ecological Patterns Are Robust to Use of Exact Sequence Variants versus Operational Taxonomic Units
Source: mSphere. 2018 Jul 18;3(4):e00148-18. doi: 10.1128/mSphere.00148-18 (PMC6052340; doi:10.1128/mSphere.00148-18)
Supplement: TABLE S1 [file sph004182596st1.docx]

**Table S1**

| Time Point | Amplicon | Kingdom | No. reads after quality filtering | No. Samples | No. OTUs | No. ESVs |
| --- | --- | --- | --- | --- | --- | --- |
| 6 months | ITS2 | Fungi | 5.9M | 95 | 1196 | 1240 |
| 12 months | ITS2 | Fungi | 7.3M | 95 | 1420 | 1594 |
| 18 months | ITS2 | Fungi | 7.3M | 114 | 1313 | 1243 |
| 6 months | 16S | Bacteria | 3.99M | 97 | 745 | 1306 |
| 12 months | 16S | Bacteria | 5.75M | 102 | 1163 | 1999 |
| 18 months | 16S | Bacteria | 5.4M | 119 | 1999 | 2513 |
