# Supplementary material for: Broadscale Ecological Patterns Are Robust to Use of Exact Sequence Variants versus Operational Taxonomic Units
Source: mSphere. 2018 Jul 18;3(4):e00148-18. doi: 10.1128/mSphere.00148-18 (PMC6052340; doi:10.1128/mSphere.00148-18)
Supplement: FIG S3 [file sph004182596sf3.docx]

**Figure S3**

A)

B)

C)
