# Supplementary material for: Broadscale Ecological Patterns Are Robust to Use of Exact Sequence Variants versus Operational Taxonomic Units
Source: mSphere. 2018 Jul 18;3(4):e00148-18. doi: 10.1128/mSphere.00148-18 (PMC6052340; doi:10.1128/mSphere.00148-18)
Supplement: TABLE S3 [file sph004182596st3.docx]

**Table S3**

| **Bacteria** | **6 months** | **OTU** |  |  |  |  |
| --- | --- | --- | --- | --- | --- | --- |
|  | Df | Sum Sq | Mean Sq | Fvalue | Pr(>F) |  |
| Site | 4 | 87281 | 21820 | 20.898 | 2.28E-11 | *** |
| Inoculum | 4 | 9952 | 2488 | 2.383 | 5.96E-02 | . |
| Site:Inoculum | 16 | 71114 | 4445 | 4.257 | 1.11E-05 | *** |
| Residuals | 70 | 73089 | 1044 |  |  |  |
|  |  |  |  |  |  |  |
| **Bacteria** | **6 months** | **ESV** |  |  |  |  |
|  | Df | Sum Sq | Mean Sq | F value | Pr(>F) |  |
| Site | 4 | 325282 | 81321 | 20.682 | 2.76E-11 | *** |
| Inoculum | 4 | 31158 | 7789 | 1.98E+00 | 1.07E-01 |  |
| Site:Inoculum | 16 | 286072 | 17880 | 4.547 | 4.33E-06 | *** |
| Residuals | 70 | 275243 | 3932 |  |  |  |
|  |  |  |  |  |  |  |
| **Bacteria** | **12 months** | **OTU** |  |  |  |  |
|  | Df | Sum Sq | Mean Sq | Fvalue | Pr(>F) |  |
| Site | 4 | 220757 | 55189 | 45.517 | < 2e-16 | *** |
| Inoculum | 4 | 25461 | 6365 | 5.25 | 0.000879 | *** |
| Site:Inoculum | 16 | 33138 | 2071 | 1.708 | 0.063351 | . |
| Residuals | 75 | 90937 | 1212 |  |  |  |
|  |  |  |  |  |  |  |
| **Bacteria** | **12 months** | **ESV** |  |  |  |  |
|  | Df | Sum Sq | Mean Sq | Fvalue | Pr(>F) |  |
| Site | 4 | 1414191 | 353548 | 37.559 | < 2e-16 | *** |
| Inoculum | 4 | 202288 | 50572 | 5.372 | 0.000737 | *** |
| Site:Inoculum | 16 | 268183 | 16761 | 1.781 | 0.049934 | * |
| Residuals | 75 | 705990 | 9413 |  |  |  |
|  |  |  |  |  |  |  |
| **Bacteria** | **18 months** | **OTU** |  |  |  |  |
|  | Df | Sum Sq | Mean Sq |  | Pr(>F) |  |
| Site | 4 | 660280 | 165070 | 134.294 | < 2e-16 | *** |
| Inoculum | 4 | 20634 | 5159 | 4.197 | 0.00407 | ** |
| Site:Inoculum | 16 | 24418 | 1526 | 1.242 | 2.59E-01 |  |
| Residuals | 74 | 90958 | 1229 |  |  |  |
|  |  |  |  |  |  |  |
| **Bacteria** | **18 months** | **ESV** |  |  |  |  |
|  | Df | Sum Sq | Mean Sq | Fvalue | Pr(>F) |  |
| Site | 4 | 2246528 | 561632 | 118.012 | <2e-16 | *** |
| Inoculum | 4 | 53361 | 13340 | 2.803 | 0.0317 | * |
| Site:Inoculum | 16 | 93870 | 5867 | 1.233 | 2.65E-01 |  |
| Residuals | 74 | 352173 | 4759 |  |  |  |

| **Fungi** | **6 months** | **OTU** |  |  |  |  |
| --- | --- | --- | --- | --- | --- | --- |
|  | Df | Sum Sq | Mean Sq | Fvalue | Pr(>F) |  |
| Site | 4 | 6163 | 1540.9 | 4.265 | 0.00377 | ** |
| Inoculum | 4 | 12633 | 3158.3 | 8.741 | 8.53E-06 | *** |
| Site:Inoculum | 16 | 13216 | 826 | 2.286 | 0.00926 | ** |
| Residuals | 71 | 25654 | 361.3 |  |  |  |
|  |  |  |  |  |  |  |
| **Fungi** | **6 months** | **ESV** |  |  |  |  |
|  | Df | Sum Sq | Mean Sq | F value | Pr(>F) |  |
| Site | 4 | 3936 | 984 | 1.763 | 0.14594 |  |
| Inoculum | 4 | 24459 | 6115 | 1.10E+01 | 5.66E-07 | *** |
| Site:Inoculum | 16 | 23398 | 1462 | 2.62 | 0.00289 | ** |
| Residuals | 71 | 39636 | 558 |  |  |  |
|  |  |  |  |  |  |  |
| **Fungi** | **12 months** | **OTU** |  |  |  |  |
|  | Df | Sum Sq | Mean Sq | Fvalue | Pr(>F) |  |
| Site | 4 | 12921 | 3230 | 7.075 | 7.64E-05 | *** |
| Inoculum | 4 | 7172 | 1793 | 3.927 | 0.00621 | ** |
| Site:Inoculum | 16 | 9612 | 601 | 1.316 | 0.21263 |  |
| Residuals | 70 | 31959 | 457 |  |  |  |
|  |  |  |  |  |  |  |
| **Fungi** | **12 months** | **ESV** |  |  |  |  |
|  | Df | Sum Sq | Mean Sq | Fvalue | Pr(>F) |  |
| Site | 4 | 10530 | 2632.6 | 3.959 | 0.00593 | ** |
| Inoculum | 4 | 6069 | 1517.3 | 2.282 | 0.06907 | . |
| Site:Inoculum | 16 | 13794 | 862.1 | 1.296 | 0.22435 |  |
| Residuals | 70 | 46550 | 665 |  |  |  |
|  |  |  |  |  |  |  |
| **Fungi** | **18 months** | **OTU** |  |  |  |  |
|  | Df | Sum Sq | Mean Sq |  | Pr(>F) |  |
| Site | 4 | 13800 | 3450 | 8.898 | 7.22E-06 | *** |
| Inoculum | 4 | 9753 | 2438 | 6.289 | 0.000221 | *** |
| Site:Inoculum | 16 | 27154 | 1697 | 4.377 | 7.50E-06 | *** |
| Residuals | 70 | 27141 | 388 |  |  |  |
|  |  |  |  |  |  |  |
| **Fungi** | **18 months** | **ESV** |  |  |  |  |
|  | Df | Sum Sq | Mean Sq | Fvalue | Pr(>F) |  |
| Site | 4 | 15399 | 3850 | 6.472 | 0.000172 | *** |
| Inoculum | 4 | 10599 | 2650 | 4.455 | 0.002894 | ** |
| Site:Inoculum | 16 | 36416 | 2276 | 3.826 | 4.63E-05 | *** |
| Residuals | 70 | 41636 | 595 |  |  |  |
