# Supplementary material for: Broadscale Ecological Patterns Are Robust to Use of Exact Sequence Variants versus Operational Taxonomic Units
Source: mSphere. 2018 Jul 18;3(4):e00148-18. doi: 10.1128/mSphere.00148-18 (PMC6052340; doi:10.1128/mSphere.00148-18)
Supplement: TABLE S4 [file sph004182596st4.docx]

**Table S4**

| **6 months** | OTU |  |  |  |  |  |  |
| --- | --- | --- | --- | --- | --- | --- | --- |
|  | Df | SumsOfSqs | MeanSqs | F.Model | R2 | Pr(>F) |  |
| Site | 4 | 5.3289 | 1.33222 | 17.4236 | 0.34467 | 1.00E-04 | *** |
| Inoculum | 4 | 2.5453 | 0.63633 | 8.3224 | 0.16463 | 1.00E-04 | *** |
| Site:Inoculum | 16 | 2.9225 | 0.18266 | 2.3889 | 0.18903 | 1.00E-04 | *** |
| Residuals | 61 | 4.6641 | 0.07646 |  | 0.30167 |  |  |
| Total | 85 | 15.4608 |  |  | 1 |  |  |
|  |  |  |  |  |  |  |  |
| **6 months** | ESV |  |  |  |  |  |  |
|  | Df | SumsOfSqs | MeanSqs | F.Model | R2 | Pr(>F) |  |
| Site | 4 | 5.7313 | 1.43282 | 12.4584 | 0.27056 | 1.00E-04 | *** |
| Inoculum | 4 | 4.0616 | 1.01539 | 8.8288 | 0.19174 | 1.00E-04 | *** |
| Site:Inoculum | 16 | 4.3744 | 0.2734 | 2.3772 | 0.20651 | 1.00E-04 | *** |
| Residuals | 61 | 7.0155 | 0.11501 |  | 0.33119 |  |  |
| Total | 85 | 21.1828 |  |  | 1 |  |  |
|  |  |  |  |  |  |  |  |
| **12 months** | OTU |  |  |  |  |  |  |
|  | Df | SumsOfSqs | MeanSqs | F.Model | R2 | Pr(>F) |  |
| Site | 4 | 6.8983 | 1.72459 | 19.7618 | 0.35664 | 1.00E-04 | *** |
| Inoculum | 4 | 2.9448 | 0.7362 | 8.436 | 0.15224 | 1.00E-04 | *** |
| Site:Inoculum | 16 | 3.216 | 0.201 | 2.3032 | 0.16627 | 1.00E-04 | *** |
| Residuals | 72 | 6.2834 | 0.08727 |  | 0.32485 |  |  |
| Total | 96 | 19.3425 |  |  | 1 |  |  |
|  |  |  |  |  |  |  |  |
| **12 months** | ESV |  |  |  |  |  |  |
|  | Df | SumsOfSqs | MeanSqs | F.Model | R2 | Pr(>F) |  |
| Site | 4 | 8.0058 | 2.00144 | 15.9659 | 0.31394 | 1.00E-04 | *** |
| Inoculum | 4 | 3.9817 | 0.99542 | 7.9406 | 0.15614 | 1.00E-04 | *** |
| Site:Inoculum | 16 | 4.4875 | 0.28047 | 2.2374 | 0.17598 | 1.00E-04 | *** |
| Residuals | 72 | 9.0257 | 0.12536 |  | 0.35394 |  |  |
| Total | 96 | 25.5007 |  |  | 1 |  |  |
|  |  |  |  |  |  |  |  |
| **18 months** | OTU |  |  |  |  |  |  |
|  | Df | SumsOfSqs | MeanSqs | F.Model | R2 | Pr(>F) |  |
| Site | 4 | 8.8405 | 2.21013 | 28.6693 | 0.4675 | 1.00E-04 | *** |
| Inoculum | 4 | 1.9093 | 0.47732 | 6.1916 | 0.10096 | 1.00E-04 | *** |
| Site:Inoculum | 16 | 2.6101 | 0.16313 | 2.1161 | 0.13802 | 1.00E-04 | *** |
| Residuals | 72 | 5.5505 | 0.07709 |  | 0.29352 |  |  |
| Total | 96 | 18.9104 |  |  | 1 |  |  |
|  |  |  |  |  |  |  |  |
| **18 months** | ESV |  |  |  |  |  |  |
|  | Df | SumsOfSqs | MeanSqs | F.Model | R2 | Pr(>F) |  |
| Site | 4 | 10.228 | 2.55701 | 21.3118 | 0.38457 | 1.00E-04 | *** |
| Inoculum | 4 | 3.3904 | 0.84761 | 7.0645 | 0.12748 | 1.00E-04 | *** |
| Site:Inoculum | 16 | 4.3388 | 0.27117 | 2.2601 | 0.16314 | 1.00E-04 | *** |
| Residuals | 72 | 8.6386 | 0.11998 |  | 0.32481 |  |  |
| Total | 96 | 26.5958 |  |  | 1 |  |  |
