# Supplementary material for: Broadscale Ecological Patterns Are Robust to Use of Exact Sequence Variants versus Operational Taxonomic Units
Source: mSphere. 2018 Jul 18;3(4):e00148-18. doi: 10.1128/mSphere.00148-18 (PMC6052340; doi:10.1128/mSphere.00148-18)
Supplement: TABLE S5 [file sph004182596st5.docx]

**Table S5**

| **6 months** | OTU |  |  |  |  |  |  |
| --- | --- | --- | --- | --- | --- | --- | --- |
|  | Df | SumsOfSqs | MeanSqs | F.Model | R2 | Pr(>F) |  |
| Site | 4 | 3.6505 | 0.9126 | 16.292 | 0.13819 | 1.00E-04 | *** |
| Inoculum | 4 | 15.1216 | 3.7804 | 67.486 | 0.57242 | 1.00E-04 | *** |
| Site:Inoculum | 16 | 3.8354 | 0.2397 | 4.279 | 0.14519 | 1.00E-04 | *** |
| Residuals | 68 | 3.8092 | 0.056 |  | 0.1442 |  |  |
| Total | 92 | 26.4167 |  |  | 1 |  |  |
|  |  |  |  |  |  |  |  |
| **6 months** | ESV |  |  |  |  |  |  |
|  | Df | SumsOfSqs | MeanSqs | F.Model | R2 | Pr(>F) |  |
| Site | 4 | 3.4677 | 0.8669 | 14.285 | 0.11983 | 1.00E-04 | *** |
| Inoculum | 4 | 17.1383 | 4.2846 | 70.599 | 0.59222 | 1.00E-04 | *** |
| Site:Inoculum | 16 | 4.2065 | 0.2629 | 4.332 | 0.14536 | 1.00E-04 | *** |
| Residuals | 68 | 4.1268 | 0.0607 |  | 0.1426 |  |  |
| Total | 92 | 28.9393 |  |  | 1 |  |  |
|  |  |  |  |  |  |  |  |
| **12 months** | OTU |  |  |  |  |  |  |
|  | Df | SumsOfSqs | MeanSqs | F.Model | R2 | Pr(>F) |  |
| Site | 4 | 3.8139 | 0.95347 | 10.747 | 0.15252 | 1.00E-04 | *** |
| Inoculum | 4 | 11.3907 | 2.84767 | 32.096 | 0.45552 | 1.00E-04 | *** |
| Site:Inoculum | 16 | 4.123 | 0.25769 | 2.904 | 0.16488 | 1.00E-04 | *** |
| Residuals | 64 | 5.6782 | 0.08872 |  | 0.22708 |  |  |
| Total | 88 | 25.0058 |  |  | 1 |  |  |
|  |  |  |  |  |  |  |  |
| **12 months** | ESV |  |  |  |  |  |  |
|  | Df | SumsOfSqs | MeanSqs | F.Model | R2 | Pr(>F) |  |
| Site | 4 | 3.6894 | 0.9223 | 9.676 | 0.13472 | 1.00E-04 | *** |
| Inoculum | 4 | 12.9589 | 3.2397 | 33.987 | 0.47322 | 1.00E-04 | *** |
| Site:Inoculum | 16 | 4.6356 | 0.2897 | 3.039 | 0.16928 | 1.00E-04 | *** |
| Residuals | 64 | 6.1006 | 0.0953 |  | 0.22278 |  |  |
| Total | 88 | 27.3845 |  |  | 1 |  |  |
|  |  |  |  |  |  |  |  |
| **18 months** | OTU |  |  |  |  |  |  |
|  | Df | SumsOfSqs | MeanSqs | F.Model | R2 | Pr(>F) |  |
| Site | 4 | 5.111 | 1.27775 | 15.693 | 0.20105 | 1.00E-04 | *** |
| Inoculum | 4 | 10.498 | 2.62451 | 32.234 | 0.41296 | 1.00E-04 | *** |
| Site:Inoculum | 16 | 4.5197 | 0.28248 | 3.469 | 0.17779 | 1.00E-04 | *** |
| Residuals | 65 | 5.2924 | 0.08142 |  | 0.20819 |  |  |
| Total | 89 | 25.4212 |  |  | 1 |  |  |
|  |  |  |  |  |  |  |  |
| **18 months** | ESV |  |  |  |  |  |  |
|  | Df | SumsOfSqs | MeanSqs | F.Model | R2 | Pr(>F) |  |
| Site | 4 | 4.7856 | 1.19641 | 13.118 | 0.17019 | 1.00E-04 | *** |
| Inoculum | 4 | 12.1426 | 3.03564 | 33.284 | 0.43183 | 1.00E-04 | *** |
| Site:Inoculum | 16 | 5.2625 | 0.32891 | 3.606 | 0.18715 | 1.00E-04 | *** |
| Residuals | 65 | 5.9283 | 0.0912 |  | 0.21083 |  |  |
| Total | 89 | 28.119 |  |  | 1 |  |  |
